# Incidence and determinants of maternal sepsis in Ghana in the midst of a pandemic

**DOI:** 10.1186/s12884-022-05182-0

**Published:** 2022-11-23

**Authors:** Charles Lwanga Noora, Adom Manu, Adolphina Addo-Lartey, Abdul Gafaru Mohammed, Donne Kofi Ameme, Ernest Kenu, Kwasi Torpey, Richard Adanu

**Affiliations:** 1 Department of Epidemiology, School of Public Health, College of Health Sciences, University of Ghana Legon, Accra, Ghana; 2 Department of Population, Family and Reproductive Health, School of Public Health, College of Health Sciences, University of Ghana Legon, Accra, Ghana

**Keywords:** Maternal sepsis, Incidence rate, Puerperal sepsis, Maternal mortality, Ghana

## Abstract

**Background:**

Despite being preventable, maternal sepsis continues to be a significant cause of death and morbidity, killing one in every four pregnant women globally. In Ghana, clinicians have observed that maternal sepsis is increasingly becoming a major contributor to maternal mortality. The lack of a consensus definition for maternal sepsis before 2017 created a gap in determining global and country-specific burden of maternal sepsis and its risk factors. This study determined the incidence and risk factors of clinically proven maternal sepsis in Ghana.

**Methods:**

We conducted a prospective cohort study among 1476 randomly selected pregnant women in six health facilities in Ghana, from January to September 2020. Data were collected using primary data collection tools and reviewing the client’s charts. We estimated the incidence rate of maternal sepsis per 1,000 pregnant women per person-week. Poisson regression model and the cox-proportional hazard regression model estimators were used to assess risk factors associated with the incidence of maternal sepsis at a 5% significance level.

**Results:**

The overall incidence rate of maternal sepsis was 1.52 [95% CI: 1.20–1.96] per 1000 person-weeks. The majority of the participants entered the study at 10–13 weeks of gestation. The study participants' median body mass index score was 26.4 kgm^−2^ [22.9—30.1 kgm^−2^]. The risk of maternal sepsis was 4 times higher among women who developed urinary tract infection after delivery compared to those who did not (aHR: 4.38, 95% CI: 1.58–12.18, *p* < 0.05). Among those who developed caesarean section wound infection after delivery, the risk of maternal sepsis was 3 times higher compared to their counterparts (aHR: 3.77, 95% CI: 0.92–15.54, *p* < 0.05). Among pregnant women who showed any symptoms 14 days prior to exit from the study, the risk was significantly higher among pregnant women with a single symptom (aHR: 6.1, 95% CI: 2.42–15.21, *p* < 0.001) and those with two or more symptoms (aHR: 17.0, 95% CI: 4.19–69.00, *p* < 0.001).

**Conclusions:**

Our findings show a low incidence of maternal sepsis in Ghana compared to most Low and Middle-Income Countries. Nonetheless, Maternal sepsis remains an important contributor to the overall maternal mortality burden. It is essential clinicians pay more attention to ensure early and prompt diagnosis. Factors significantly predicting maternal sepsis in Ghana were additional maternal morbidity, urinary tract infections, dysuria, and multiple symptoms. We recommend that Ghana Health Service should institute a surveillance system for maternal sepsis as a monthly reportable disease.

**Supplementary Information:**

The online version contains supplementary material available at 10.1186/s12884-022-05182-0.

## Background

Maternal sepsis is a life-threatening condition defined as organ dysfunction resulting from infection during pregnancy, childbirth, post-abortion, or postpartum period” [1]. It is estimated that sepsis contributes to more than 100,000 of all maternal deaths annually, but primarily responsible for more than 35,000 deaths [2]. In Africa, maternal sepsis is estimated to cause about 9.7% of all maternal deaths [3, 4]. A nationwide secondary data analysis of autopsy and maternal mortality data indicates maternal sepsis is the fourth leading cause of maternal deaths accounting for 9.1% of all maternal deaths [5]. Maternal sepsis is increasingly becoming a major contributor to maternal mortality in Ghana [5, 6].

Pregnancy, labor, and following childbirth present a period of high risk for infections leading to maternal sepsis. There is generally immune suppression during these periods due to tissue and physiological changes, particularly during labor and immediately after childbirth in the placental bed of the uterus (Acosta & Knight, 2013). Unfortunately, this period creates a conducive environment for microorganisms to thrive. Additionally, some heath practices; including self-medication may further lower their immunity whiles interventions such as vaginal examinations could also introduce microbes leading to infections. The spectrum of infections during pregnancy and following childbirth ranging from mild infection to severe sepsis and septic shock causes high mortality, longer hospitalizations, and excessive cost to the patients and healthcare system.

Despite being preventable, maternal sepsis continues to be a major cause of death and morbidity for pregnant women, killing one in every four pregnant women [7–9]. Improving maternal health is a crucial priority for the WHO, culminating in the adoption of a global strategy and goal of ending preventable maternal mortality. The main strategy is to address all causes of maternal mortality, reproductive and maternal morbidities in all WHO countries.

Earlier definitions of sepsis during and following pregnancy was; a temperature rise above 100.4 F (38 °C) maintained over 24 h or recurring during the period from the end of the first to the end of the 10th day after childbirth or abortion [10], *Puerperal sepsis* is an infection of the genital tract occurring at any time between the rupture of membranes or labour and the 42nd day postpartum, in which, two or more of the following are present: pelvic pain, fever, abnormal vaginal discharge and delay in the reduction of the size of the uterus [11]. *Systemic inflammatory response syndrome (SIRS):* Widespread inflammatory response to severe clinical insult defined by the presence of two or more of the following symptoms: Temperature > 38 °C or < 36 °C, Heart rate > 90/min, Respiratory rate > 20/min or PaCO_2_ < 32 mmHg, white blood cells > 12 × 10^9/dL or < 4 × 10^9/dL or > 10% immature forms. *Sepsis*: SIRS plus definitive evidence of infection. *Severe sepsis*: Sepsis with signs of organ dysfunction, hypoperfusion or hypotension.

*Septic shock*: Sepsis with hypotension despite adequate fluid resuscitation (The American College of Chest Physicians and the Society of Critical Care Medicine [12]

The lack of a consensus definition for maternal sepsis before 2017 created a gap in the determination of global and country-specific burden of maternal sepsis. Ghana is one of 52 WHO member countries that validated the new definition of maternal sepsis and has since employed the new definition in its diagnosis. There has never been an opportune time to document the burden and identify risk factors along the continuum of care for pregnant women and following delivery according to a global consensus definition, identification, and classification of maternal sepsis than now. Prior to the study there was little knowledge on the incidence of maternal sepsis among women in the different parts of the country. Therefore, this study sought to determine the incidence and identified risk factors of clinically proven maternal sepsis among a cohort of pregnant women in Ghana.

## Methods

### Study design

This was a prospective cohort study among pregnant women in three regions of Ghana from 01 January 2020 and 30 September 2020. Women attending antenatal care (ANC) at booking (first registration) regardless of gestational age of the pregnancy, were randomly recruited and followed until delivery and six weeks after delivery. The attending midwife or clinician throughout the study period screened women using the WHO new case definition of maternal sepsis. Data was quantitatively collected through interviews, records review and physical assessments.

### Study setting

Ghana is a West African country broadly zoned into three; Coastal, middle and Northern zones and administratively divided into 16 regions with 260 districts. There are regional hospitals in each of the 16 regions with supporting district and community level health facilities. To ensure all three zones of the country was well represented based on available resources, the research was carried out in three out of 16 regions (one from each zone) in Ghana with a total population of more than 7 million people. The Greater Accra Region, Bono Region, and Upper West Region were chosen. Data was collected at six health care facilities across the three regions.

Ghana's population is estimated to be 30.8 million, with a yearly growth rate of 2.4 percent, according to the Ghana Statistical Service (GSS) (GSS, 2021).

**Fig. 1:**
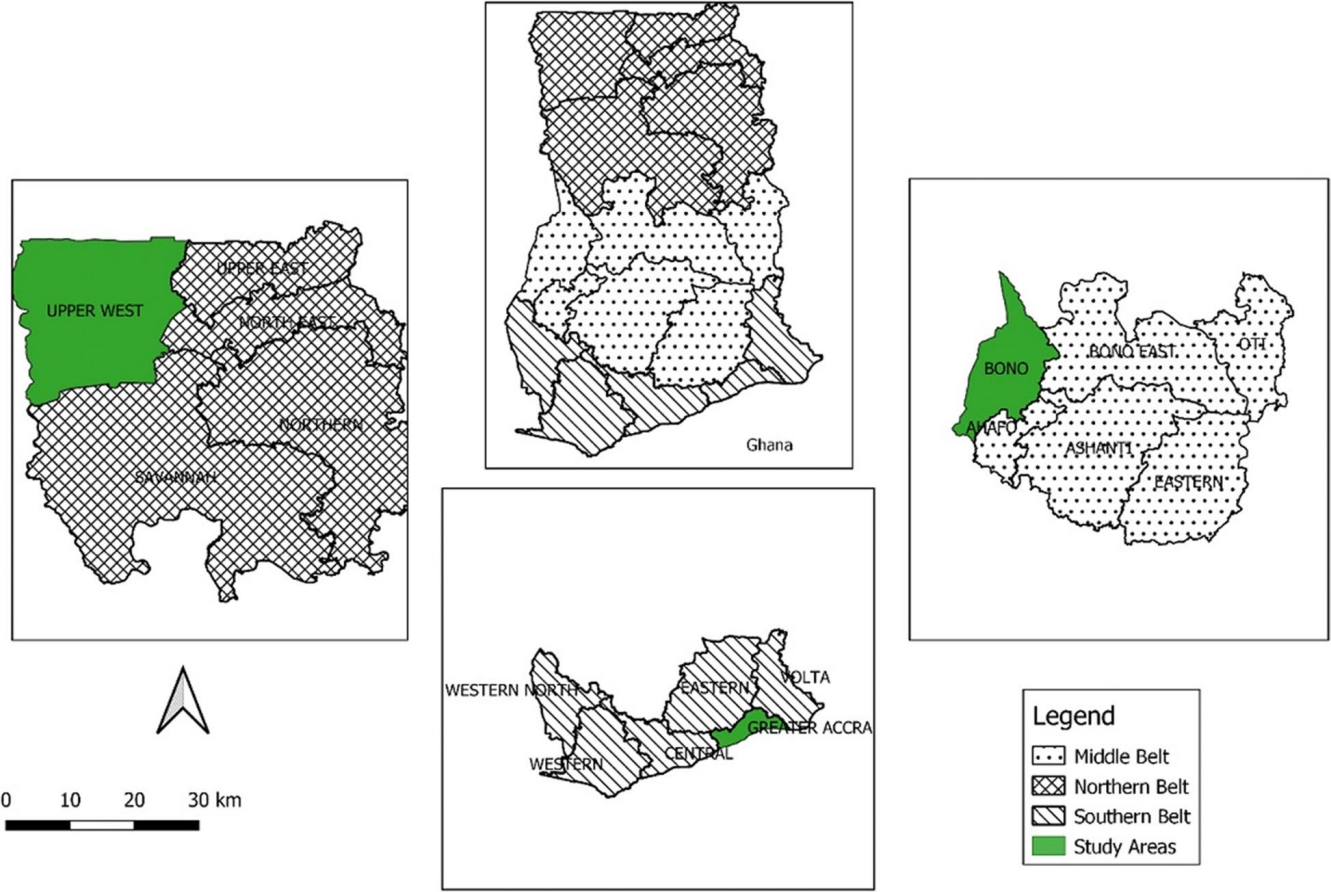
Selected Regions from the three epidemiological zones, Ghana, 2022

The data collection sites included; Wa Municipal Hospital, Jirapa Municipal, Hospital, Sunyani Municipal Hospital, Bono regional Hospital, Tema General Hospital and Greater Accra Regional Hospital (Ridge). The Greater Accra Regional hospital is a 420-bed capacity hospital in the Osu-Klotey sub metro in the Accra metropolitan area that provides inpatient and outpatient services and specialized services. On average, there are 154,098 ANC registrants in GAR with more than 752, 000 ANC attendance and some 112, 000 deliveries. The Brong Ahafo Regional Hospital is a 330-bed capacity health facility that provides OPD and inpatient care services. Sunyani Municipal Hospital is a 95-bed capacity hospital located in the eastern part of the Sunyani Municipality that provides Outpatient and Inpatient services. Average ANC registrants in the Bono Region stands at 35,868 with more than 205, 000 ANC attendance and some 31, 000 deliveries. The Wa Municipal Hospital and Jirapa Hospital each is a 200-bed capacity facility in the Upper West region which provides both OPD and Inpatient services. The Average ANC registrants and attendance in the UWR are 27. 784 and 137,648 respecitvely and some 23,893 deliveries (Fig. 1).

### Inclusion criteria

All pregnant women regardless of age who were registered in antenatal clinics of the selected health facilities, and who did not intend to leave the study site over the study period were included.

### Exclusion criteria

Records of women who become pregnant with assistance of advanced reproductive technology, women with pre-existing medical conditions outside of pregnancy (e.g., HIV positive, hepatitis B or hepatitis C, hypertension, renal disease) at study enrolment were excluded.

### WHO definition of maternal sepsis

In 2017, the definition of maternal sepsis was updated. WHO now defines maternal sepsis as “organ dysfunction resulting from infection during pregnancy, childbirth, post-abortion, or postpartum period”, which includes 42 days after the pregnancy has terminated, irrespective of the cause [1].The disease-specific criteria set by WHO [13] were employed to identify sepsis in pregnancy and following child birth including maternal near-miss cases.

### Sample size estimation

Epi info version 7 Statcalc (Fleiss) was used to determine the minimum sample size for this study cohort. Using a 95% confidence level, power of 80% and expected increase in the incidence of maternal sepsis by 6% at the end of the study, a minimum sample size of 245 was estimated for each of the six districts, given a total minimum sample size of 1470. Each district's sample was treated separately as coming from an independent population without any stratification.

### Study eligibility and sampling process

We used a multistage sampling approach to select 1476 pregnant women for the study. The first stage involved the selection of three regions from the three epidemiological zones in Ghana. The Upper West region in the Northern Zone, Bono region in the Middle Zone, and Greater Accra region in the Coastal Zone were randomly selected respectively. The second phase involved the sampling of health facilities, one district hospital was then randomly selected from each of the three regions in addition to the regional hospital. A proportionate to size sampling approach using the ANC attendance rate in each of the selected health facilities was done. Pregnant women who met the selection criteria were randomly selected at ANC exit points and recruited into the study after obtaining written informed consent.

### Data collection process

Data was collected by trained research assistants (nurses) selected from the selected health facilities through in-person assessment. A scheduled follow-up exercise was carried out in collecting data from the study participants. All the recruited women were followed up until delivery and 6 weeks after delivery. The follow-up period was in three phases using a follow-up log book. Phase One was from booking to labor, the follow-up was bi-weekly, Phase two was daily from delivery till discharge from the health facility or seven days after delivery. The third phase was bi-weekly after discharge or seven days after delivery until 42 days after delivery or death.

Data was collected on demographic characteristics, obstetric characteristics, anthropometric measurements, health status (clinical diagnosis or symptoms 14 days prior to diagnosis) and health facility characteristics.

Demographic and Obstetric characteristics collected included place of residence, age, occupation, educational level, income level, religion, marital status, parity, gravida, initiation of ANC and number of ANC attendance.

Anthropometric measurements: Weight and height were measured at study enrolment (antenatal booking) using a standard instrument (SECA digital weighing scale, SECA body meter, and SECA measuring tape). Body weight (current pregnancy) and weight at the delivery were obtained from medical records and BMI was calculated as weight (kilogram) divided respondents’ height (square meters).

The independent variables were extracted from the maternal records on demographic characteristics, clinical and obstetric history and the availability of specialist medical doctor (yes or No).

### Outcome measures

The outcome variable “maternal sepsis” was measured using the 2017 World Health Organization experts’ definition [13, 14]. Thus, maternal sepsis was diagnosed as ‘yes’ when diagnosed by the clinician and ‘no’ when clinician did not suspect infection during hospital visit, hospitalization or follow up visits post-delivery.

### Data management and statistical analysis

Data collected was cleaned and exported to Stata version 16 for analysis. The background characteristics of study participants were described using frequencies and percentages for categorical variables, means and standard deviation for normally distributed continuous variables and median and interquartile range for non-normal continuous variables. The incidence rate of maternal sepsis per 1,000 pregnant women per person-week was also estimated.

Poisson regression model estimator and the cox-proportional hazard regression model estimators were used to assess risk factors associated with the incidence of maternal sepsis at a 5% level of significance. For each of the two estimators, two different regression models were fitted. Model 1 of each of the two estimators was populated with the independent variables that were significantly associated with maternal sepsis from the crude incidence rate ratio estimates. Whilst model 2 of each of the two estimators contained all of the independent variables considered in this study. The Poisson regression model estimated the adjusted incidence rate ratio whilst the cox-proportional hazard model estimated the hazard rate ratio. The log-likelihood ratio test was used to assess the most appropriate models at 5% significance level.

### Ethical consideration

Prior to the commencement of the study, ethical approval was obtained from the Ghana Health Service Ethical Review Committee (GHS-ERC011/10/19). Permission letters were obtained from Regional Health Directorates and Participating Health facilities before starting the study. Written informed consent and or parental assent was obtained from participants of the study without any form of coercion.

## Results

### Characteristics of study participants, Ghana, 2022

A total of 1476 women were used for the study's final analysis. The mean age of the women was 29.0 years with a standard deviation of 6.0 years. Eight of every ten of the women were married (80.8%). About a tenth of the women had no formal education (9.7%), 8.8% had primary education, 30.4% had Junior High School (JHS) level education, 24.9% were with Senior High School (SHS) education and 26.2% had tertiary level education. About two-thirds of the participants entered the study at 10–13 weeks of gestation, a third at 6–9 weeks gestation, and 2.4% at less than 6 weeks gestation. The study participants' median body mass index score was 26.4 kgm^−2^ with an inter-quartile range of 22.9 to 30.1 kgm^−2^. At the end of their pregnancies, the participants' mean number of antenatal care attendance was 5 visits with a quarter attaining less than 4 ANC visits, about two-thirds attaining 4 to 7 ANC visits, and 11.5% attaining 8 or more ANC visits (Table 1).

**Table 1:** Descriptive characteristics of study participants, Ghana, 2022

Variables & categories	Frequency (*N* = 1476)	Percentage (%)
**Age at booking (years), (mean ± SD)**	29.0 ± 6.0	
< 20	74	5.0
20–35	1192	80.8
> 35	210	14.2
**Marital status**		
Not married	266	18.0
Married	1192	80.8
**Highest educational level**		
No formal education	143	9.7
Primary	130	8.8
JHS	448	30.4
SHS	368	24.9
Tertiary	387	26.2
**Religion**		
Christians	1174	79.5
Islam	287	19.4
Others	15	1.0
**Main occupation**		
Unemployed	198	13.4
Formal sector	234	15.9
Informal sector	1044	70.7
**BMI category, median (IQR)**	26.4 (22.9, 30.1)	
< 18.5kgm2	39	2.6
18.5 to 24.9kgm2	530	35.9
25.0 to 29.9kgm2	531	36.0
30.0 to 34.9kgm2	262	17.8
> 34.9kgm2	114	7.7
**Gravidity, median (IQR)**	2.0 (1.0, 4.0)	
1 pregnancy	373	25.3
2–3 pregnancies	678	46.0
> 3 pregnancies	424	28.7
**Parity, median (IQR)**	1.0 (0.0, 2.0)	
No child	522	35.4
1–2 children	706	47.8
> 2 Children	248	16.8
**Number of ANC visits, mean ± SD**	5.1 ± 2.1	
< 4 visits	371	25.1
4 to7 visits	935	63.3
8 + visits	170	11.5
**Type of health facility**		
Regional hospital	735	49.8
District hospital	741	50.2
**Region**		
Bono	469	31.8
Greater Accra	592	40.1
Upper West	415	28.1
**Mode of delivery**		
SVD	987	68.0
CS	465	32.0
**Birth attendant**		
Obstetrician/Physician	439	30.0
Midwife/Nurse	1011	69.2
Others (TBA/family member)	12	0.8

**Fig. 2:**
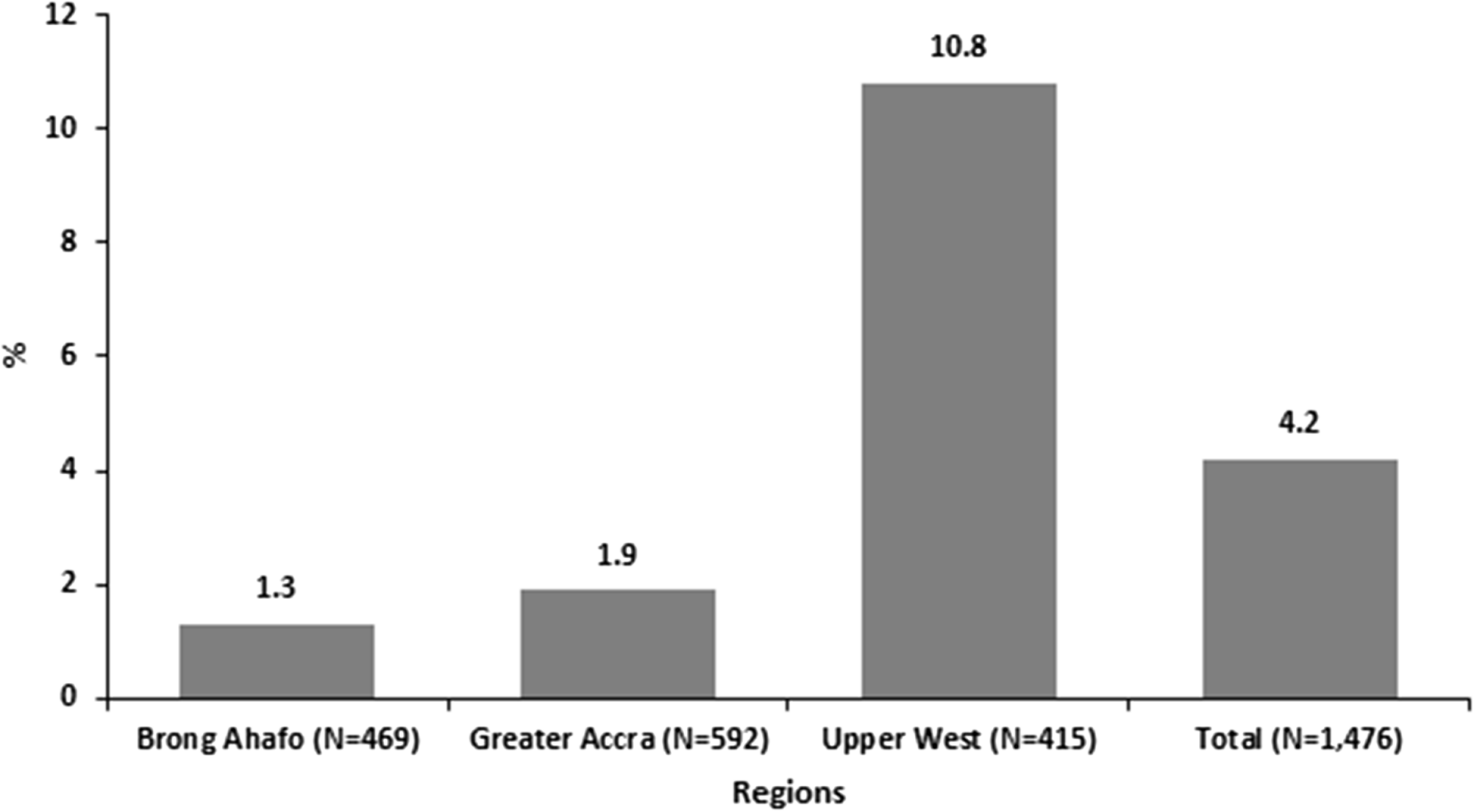
Cumulative Incidence of sepsis among study participants by region, Ghana, 2022

At the exit of the study, 62 out of the 1,476 total study participants were diagnosed with maternal sepsis representing 3.7%. At the end of the study, the 1,476 study participants contributed 40,934 persons-weeks. A total of 62 new cases of maternal sepsis meant that the incidence rate was 1.52 maternal sepsis cases per person-week per 1,000 women. Majority, 84% (52/62) of them developed sepsis within 14 days prior to delivery. A 95% confidence interval estimate of maternal sepsis was estimated from 1.20 to 1.96 incidence of maternal sepsis per person-week per 1000 women (Fig.2).

### Incidence and incidence rate ratio of maternal sepsis by background characteristics of study participants, Ghana, 2022

The incidence of maternal sepsis was 54% lower among pregnant women with 2–3 pregnancies compared to those with single pregnancy (IRR: 0.51, 95% CI: 0.30–0.89, *p* < 0.01). Overall, the gravidity of the pregnant women was significantly associated with the incidence of maternal sepsis from the crude estimates (*p* = 0.003). The rate of maternal sepsis was over 5 times higher among women who visited facilities with no specialists available (IRR: 5.28, 95% CI: 2.81–9.91, *p* < 0.001). Compared to women from the Bono region, the incidence rate of maternal sepsis was over 8 times significantly higher in the Upper West region (IRR: 8.19, 95% CI: 3.49–19.20, *p* < 0.001) (Table 2).

**Table 2:** Incidence and incidence rate ratio of maternal sepsis by background characteristics of study participants, Ghana, 2022

	Total person, time in weeks	Number of incidences	Incidence rate per week per 1000 women	Unadjusted Poisson regression model	
				IRR [95% CI]	*P*-value
Overall	40,832.4	62	1.52 [1.20–1.96]		
Age at booking (years)					0.234
< 20	2042.4	3	1.47 [0.47–6.92]	1.00	
20—35	33,000.3	55	1.67 [1.29–2.18]	1.13 [0.35–3.63]	
> 35	5789.7	4	0.69 [0.26–2.45]	0.47 [0.11–2.10]	
Marital status					0.350
Not married	7844.0	9	1.15 [0.61–2.41]	1.00	
Married	32,988.4	53	1.61 [1.24–2.12]	1.40 [0.69–2.84]	
Highest education					0.862
None/Primary	7650.4	13	1.70 [1.02–3.07]	1.00	
JHS	12,351.4	16	1.30 [0.81–2.20]	0.76 [0.37–1.58]	
SHS	10,109.7	17	1.68 [1.07–2.80]	0.99 [0.48–2.04]	
Tertiary	10,720.9	16	1.49 [0.94–2.53]	0.88 [0.42–1.83]	
Religion					< 0.001***
Christians	32,371.0	35	1.08 [0.79–1.53]	1.00	
Islam/others	8461.4	27	3.19 [2.25–4.69]	2.95 [1.79–4.88] ***	
Main occupation					0.439
Unemployed	5483.0	11	2.01 [1.15–3.83]	1.00	
Formal sector	6467.7	7	1.08 [0.53–2.57]	0.54 [0.21–1.39]	
Informal sector	28,881.7	44	1.52 [1.15–2.07]	0.76 [0.39–1.47]	
BMI category					0.133
< 18.5kgm2	1103.1	4	3.63 [1.46–11.81]	1.00	
18.5 to 24.9kgm^−2^	14,814.7	24	1.62 [1.11–2.47]	0.45 [0.16–1.29]	
25.0 to 29.9kgm^−2^	14,247.6	24	1.68 [1.15–2.57]	0.46 [0.16–1.34]	
> 29.9kgm^−2^	10,667.0	10	0.94 [0.52–1.88]	0.26 [0.08–0.82] *	
Gravidity					0.004**
1 pregnancy	10,385.4	27	2.60 [1.83–3.84]	1.00	
2–3 pregnancies	18,704.0	25	1.34 [0.92–2.02]	0.51 [0.30–0.89] *	
> 3 pregnancies	11,719.1	10	0.85 [0.47–1.71]	0.33 [0.16–0.68] **	
Parity					0.152
No child	14,400.6	29	2.01 [1.43–2.94]	1.00	
1–2 children	19,549.0	23	1.18 [0.80–1.82]	0.58 [0.34–1.01]	
> 2 children	6882.9	10	1.45 [0.81–2.90]	0.72 [0.35–1.48]	
Number of ANC visits					0.354
< 4 visits	10,370.7	12	1.16 [0.67–2.16]	1.00	
4 to 7 visits	25,799.0	40	1.55 [1.15–2.13]	1.34 [0.70–2.55]	
8 + visits	4662.7	10	2.14 [1.19–4.26]	1.85 [0.80–4.29]	
Type of health facility					0.001**
Regional hospital	20,435.4	44	2.15 [1.63–2.91]	1.00	
District hospital	20,397.0	18	0.88 [0.57–1.45]	0.41 [0.24–0.71] **	
Availability of specialist in facility					< 0.001***
Specialist available	22,821.3	12	0.53 [0.31–0.99]	1.00	
No specialist available	18,011.1	50	2.78 [2.14–3.67]	5.28 [2.81–9.91] ***	
Region					< 0.001***
Bono	12,840.7	6	0.47 [0.21–1.22]	1.00	
Greater Accra	16,232.4	11	0.68 [0.38–1.31]	1.45 [0.54–3.92]	
Upper West	11,759.3	45	3.83 [2.92–5.12]	8.19 [3.49–19.20] ***	
Mode of delivery					0.669
SVD	27,293.4	43	1.58 [1.18–2.14]	1.00	
CS	12,884.4	18	1.40 [0.90–2.29]	0.89 [0.51–1.54]	
Birth attendant					0.794
Obstetrician/Physician	12,208.7	18	1.47 [0.95–2.42]	1.00	
Midwife/Nurse	27,903.1	43	1.54 [1.16–2.10]	1.05 [0.60–1.81]	
Others (TBA/family member)	337.7	1	2.96 [.-.]	2.01 [0.27–15.04]	

### Pregnancy and other related outcomes among mothers with sepsis

Among the 62 maternal sepsis cases recorded, none of them resulted in maternal death or stillbirth. Delivery through caesarean section was 29.0%, with 61.1% of the CS delivery being emergency cases. Admission to ICU was 40.3%. Most of the maternal sepsis cases were diagnosed upon admission as infections (41.9%). Majority (64.5%) were hypertension related complications. All but one of the 40 hypertension related complications cases were proteinuria (97.5%), epigastric pain (97.5%), headache (97.5%), pulmonary Oedema (97.5%) and visual symptoms (97.5%) (Table 3).

**Table 3:** Pregnancy and other related outcomes among mothers with sepsis

Factor	Maternal sepsis (*N* = 62)
**Maternal death**	0 (0.0)
**Abortion/ ectopic pregnancy**	6 (9.7)
**Still birth**	0 (0.0)
**Caesarean section (CS) delivery**	18 (29.0)
**Type of CS delivery (*N* = 18)**	
*Planned*	7 (38.9)
*Emergency CS*	11 (61.1)
**Admission to ICU**	25 (40.3)

### Multivariate regression models of risk factors associated with incidence of sepsis among pregnant women

From the multivariable Cox-proportional regression model 2, the risk of maternal sepsis was 88% lower among women who had more than 3 pregnancies (aHR: 0.12 95% CI: 0.02–0.95, *p* < 0.05).

The risk of maternal sepsis was 4 times higher among women who developed urinary tract infection after delivery compared to those who did not (aHR: 4.38, 95% CI: 1.58–12.18, *p* < 0.05).

Among women who developed caesarean section wound infection after delivery, the risk of maternal sepsis was 3 times higher (aHR: 3.77, 95% CI: 0.92–15.54, *p* < 0.05).

Relative to pregnant women who had no symptoms present 14 days before exit from study, the risk of maternal sepsis was significantly higher among pregnant women with a single symptom (aHR: 6.1, 95% CI: 2.42–15.21, *p* < 0.001) and those with 2 or more symptoms (aHR: 17.0, 95% CI: 4.19–69.00, *p* < 0.001) (Table 4).

**Table 4:** Multivariate regression models of risk factors associated with incidence of sepsis among pregnant women

	**Cox-proportional hazard model**	
	**Model 3**	**Model 4**
**variables & categories**	**aHR [95% CI]**	**aHR [95% CI]**
**Age at booking**	0.98 [0.92, 1.04]	0.97 [0.91, 1.04]
**Gravidity**		
1 pregnancy	1.00	1.00
2–3 pregnancies	0.92 [0.48, 1.76]	0.75 [0.22, 2.55]
> 3 pregnancies	0.52 [0.21, 1.30]	0.12 [0.02, 0.95] *
**Parity**		
No child		0.18 [0.02, 1.37]
1–2 children		0.20 [0.03, 1.11]
> 2 children		1.00
**Marital status**		
Not married		1.00
Married		0.79 [0.34, 1.86]
**Highest education**		
Primary		1.00
JHS		1.44 [0.54, 3.84]
SHS		1.05 [0.41, 2.70]
Tertiary		1.36 [0.53, 3.48]
**Religion**		
Christians	1.00	1.00
Islam/others	1.12 [0.58, 2.18]	1.16 [0.56, 2.37]
**BMI category**		
< 18.5kgm2	1.00	1.00
18.5 to 24.9kgm2	0.91 [0.25, 3.35]	0.87 [0.21, 3.57]
25.0 to 29.9kgm2	1.90 [0.49, 7.44]	2.42 [0.55, 10.54]
30.0 to 34.9kgm2	1.27 [0.29, 5.56]	1.18 [0.24, 5.75]
**Main occupation**		
Unemployed		1.00
Employed		1.69 [0.70, 4.11]
**Number of ANC visits**		
< 4 visits		1.00
4 to 7 visits		0.88 [0.42, 1.86]
8 + visits		3.49 [1.28, 9.51] *
**Type of health facility**		
Regional hospital	1.00	1.00
District hospital	0.47 [0.21, 1.02]	0.43 [0.18, 1.01]
**Availability of specialist in facility**		
Specialist available	1.00	1.00
No specialist available	8.37 [0.78, 89.91]	7.57 [0.65, 87.62]
**Region**		
Bono	1.00	1.00
Greater Accra	4.63 [0.55, 38.75]	5.65 [0.65, 48.98]
Upper West	2.64 [0.85, 8.25]	3.20 [0.94, 10.86]
**Mode of delivery**		
SVD		1.00
CS		0.84 [0.40, 1.77]
**Birth attendant**		
Obstetrician/Physician		1.00
Midwife/Nurse		1.14 [0.55, 2.36]
Others (TBA/family member)		6.35 [0.52, 78.18]
**Clinical diagnosis of infection**		
Mastitis	1.14 [0.39, 3.29]	1.48 [0.50, 4.38]
CS wound infection	2.34 [0.71, 7.72]	3.77 [0.92, 15.54]
Urinary tract infection	4.19 [1.68, 10.47] **	4.38 [1.58, 12.18] **
malaria	1.54 [0.60, 3.93]	1.38 [0.49, 3.88]
Other infection	4.24 [1.13, 15.89] *	10.55 [2.33, 47.81] **
**Symptoms 14 days prior to diagnosis**		
Abdominal pain excluding contractions	2.58 [1.05, 6.33] *	1.48 [0.49, 4.40]
Abnormal vagina discharge	0.55 [0.24, 1.24]	0.64 [0.26, 1.56]
Sore throat /cough		0.52 [0.14, 1.97]
Chest pain	0.80 [0.30, 2.11]	1.14 [0.35, 3.64]
Dysuria	0.26 [0.09, 0.75] *	0.22 [0.07, 0.71] *
Vomiting/ diarrhoea		0.24 [0.08, 0.77] *
Flu-like symptoms		0.67 [0.20, 2.28]
**Number of different symptoms 14 days prior to study**		
None	1.00	1.00
One	5.93 [2.59, 13.58] ***	6.06 [2.42, 15.21] ***
Two or more	8.54 [2.98, 24.42] ***	17.00 [4.19, 69.00] ***

**NOTE:**

Model 1: all variables significant from the unadjusted incidence rate ratios

Model 2: All independent variables in the study

*CI* Confidence interval, *aRR* Adjusted risk ratio, *aHR* Adjusted hazard ratio

*P*-value definition: *p* < 0.05*, *p* < 0.01**, *P* < 0.001***

Other infections refers to bacterial infections including syphilis, gastritis

Maternal sepsis remains a major public threat to women's survival during pregnancy and through delivery globally despite improvements in diagnosis. This is particularly worse for LMICs including Ghana where there is a triple disease (infectious, non-communicable diseases and RTAs) burden. The lack of a consensus definition for maternal sepsis before 2017 created an even more serious problem allowing all forms of categorization of women as having sepsis. This created a gap in a lack of global and country-specific burden of maternal sepsis. The findings from this study show an overall incidence of 1.52/1000 women per week of suspected or confirmed maternal sepsis in pregnant or recently pregnant women. The findings from this study are similar to earlier studies although without a standardized Case definition on maternal sepsis and puerperal sepsis in sub-Saharan Africa. In Benin, studies by Filippi et al. [15] found the incidence of maternal sepsis rate to be 0.4/1000 pregnant women similar to a study in Senegal where de Bernis et al. [16] in a population-based cohort study in two regions found the incidence of maternal sepsis to be 0.2/10000 women. Like-wise similar studies in southern Africa by Vandecruys et al. [17] found the incidence of maternal sepsis to be 0.7 per 10, 000 women. In a cross-sectional study in six countries in West Africa, incidents of maternal sepsis have also been found to be low, less than 0.1/10000 women [18]. On the contrary, studies conducted in Uganda and Benin reported a relatively higher incidence of maternal sepsis compared to the findings from this study [19, 20]. The variation in incidence rates of maternal sepsis reported by various studies from sub-Saharan Africa could be due to different contexts/settings, variation in the criteria used to define maternal sepsis, or rigor used to carry out the study.

This study found that Urinary Tract Infections (UTIs) prior to diagnosis were identified as a significant factor of maternal sepsis among women in Ghana. Interestingly we also realize that women diagnosed with other bacterial infections 14 days prior to the study had a significantly higher risk for maternal sepsis. The risk was five folds compared to women without a history of bacterial infections. Similar observations were made in Tanzania, which reported a significant association between bacterial infections and puerperal sepsis [21]. This outlines the need to effectively monitor the cardinal signs and symptoms in pregnancy, through childbirth, and during the postpartum period. This can help us diagnose early and employ the appropriate interventions or treatments for the affected women.

The study found that women with prior history of caesarean sections (CS) wound infections were at a higher risk of maternal sepsis though not statically significant and could be due to chance or is biological plausible. This could increase a woman's risk of maternal sepsis by more than four times. Therefore, health professionals should pay more attention to women who have prior CS wound during antenatal visits. This is consistent with previous studies that found a higher risk of maternal sepsis among women with a history of CS [21, 22]. On the other hand, a similar study found a lower odds of maternal sepsis associated with CS among Ethiopian women as compared to spontaneous vaginal delivery [23].

Deprived health facilities (rural regions) contributed significantly to women developing maternal sepsis as this can restrict the access and utilization of ANC and other obstetric services. Residence affects maternal health outcomes. Women who reside in urban communities could be associated with access to better learning opportunities, better financial status, better birth spacing, better accessibility to vital health information and services [24].

This study found multiple ANC visits (8 or more visits) to significantly influence clinician’s index of suspicion of pregnant women for mater sepsis. This may be explained by two possibilities, the frequency of the visit was probably because the women were indeed sick and most likely, and due to sepsis or due to a confounding factor (co-morbidities) that could be explored in future studies. Additionally, in the Ghanaian context of health delivery system, this could be explained by the nature of the operational structures of our health system due to lack of adequate health personnel resulting in unequitable distributions of same. This has the tendency of poor differential diagnosis at the lower levels before a qualified professional sees such clients for an appropriate diagnosis. Findings from the study indicate a lot more is required of our clinicians and midwives to correctly and appropriately, implement the focal antenatal care policy. Through the focal antenatal care mothers will be properly diagnosed and educated of early warning signs of sepsis and UTIs. Mothers with better education particularly hand hygiene will improve maternal health and reduce infections in pregnancy. The findings from this longitudinal study also highlight areas for future research. Similar studies should be conducted to capture the entire period of the continuum of care for pregnant women to generate further population-based data on the burden and management of maternal sepsis. The effect of essential neonatal care on late-onset infections also needs to be examined.

A few limitations were however, identified, the exclusion of HIV-positive women and pregnant women who were likely to drop out of study or complete follow up due their lack of commitment to a single facility resulting from stigma and also their reduced immunity that increases their risk to infections from the study could have influenced the incidence of maternal sepsis. For this reason, we may not be adequately representing the disease in these minority population groups. Also, the lack of laboratory capacity across all study centers resulted in no available laboratory data on specific diagnoses and to confirm organ dysfunctions. Thus, we could not confirm clinician suspicion of sepsis. However, the new WHO definition allows for clinically diagnosed maternal sepsis, considering the lack of capacity in LMICs including Ghana. Also, the COVID-19 pandemic also reduced the number of women reporting to the various health facilities. This was likely to influenced the incidence rate.

## Conclusions

Our findings show a low incidence of maternal sepsis in Ghana compared to most Low and Middle-Income Countries. Nonetheless, Maternal sepsis remains an important contributor to the overall maternal mortality burden. It is essential clinicians pay more attention to ensure early and prompt diagnosis. Factors significantly predicting maternal sepsis in Ghana were additional maternal morbidity, urinary tract infections, dysuria, and multiple symptoms. We recommend that Ghana Health Service should institute a surveillance system for maternal sepsis as a monthly reportable disease.

## Supplementary Information

**Additional file 1** 

## Data Availability

The datasets used and/or analysed during the current study are available from the corresponding author on reasonable request.
