# Supplementary material for: Incidence and determinants of maternal sepsis in Ghana in the midst of a pandemic
Source: BMC Pregnancy Childbirth. 2022 Nov 23;22:864. doi: 10.1186/s12884-022-05182-0 (PMC9686217; doi:10.1186/s12884-022-05182-0)
Supplement: Supplementary file 1 — Additional file 1 [file 12884_2022_5182_MOESM1_ESM.pdf]

Case definitions of maternal infections

Suspected infection:
A woman (with suspected infection) has “Infections WITH complications” IF: The woman has any of the following (to treat the source of infection): Laparatomy and lavage, Incision and drainage, Hysterectomy, Vacuum aspiration, Percutaneous drainage, Wound debridement, Culdotomy/Colpotomy/Culdocentesis, Dilatation and curettage, Removal of infected cannula/line, Any other surgery
OR
The woman has any of the following condition:
Shock, Cardiac Arrest, Severe hypoperfusion, Severe acidosis, Use of continuous vasoactive drugs, Cardio-pulmonary resuscitation, Acute cianosis, Gasping, Severe tachypnea, Severe bradipnea, Severe hipoxemia, Intubation and ventilation not related to anaesthesia, Oliguria non responsive to fluids or diuretic, Severe acute azotemia, Dialysis for acute renal failure, Failure to form clots, Severe acute thrombocytopenia, ive transfusion of blood or red cells, Jaundice in the presence of preeclampsia, Severe acute hyperbilirubinemia, Prolonged unconsciousness or coma, Stroke, Uncontrollable fit/status epilepticus, global paralysis, Hysterectomy due to uterine infection or haemorrhage
OR
Woman was admitted to ICU or high dependency care
OR
Woman was transferred to another facility
OR
Related infection death
Otherwise, the woman with suspected infection has “Infections WITHOUT complications”
Infection with complication:
A woman with an infection with complications has “Severe maternal outcomes related to infection (SMO)” IF: The woman has any of the following condition:
Shock, Cardiac Arrest, Severe hypoperfusion, Severe acidosis, Use of continuous vasoactive drugs, Cardio-pulmonary resuscitation, Acute cianosis, Gasping, Severe tachypnea, Severe bradipnea, Severe hipoxemia, Intubation and ventilation not related to anaesthesia, Oliguria non responsive to fluids or diuretic, Severe acute azotemia, Dialysis for acute renal failure, Failure to form clots, Severe acute thrombocytopenia, ive transfusion of blood or red cells, Jaundice in the presence of preeclampsia, Severe acute hyperbilirubinemia, Prolonged unconsciousness or coma, Stroke, Uncontrollable fit / status epilepticus, Global paralysis, Hysterectomy due to uterine infection or haemorrhage
OR
Related infection death
Otherwise, the woman (with an infection with complications) has “Non severe maternal outcomes related to infection (NON SMO)”

SMO:
A woman with a SMO has an “Infection related Near Miss” IF: The woman has any of the following condition:
Shock, Cardiac Arrest, Severe hypoperfusion, Severe acidosis, Use of continuous vasoactive drugs, Cardio-pulmonary resuscitation, Acute cianosis, Gasping, Severe tachypnea, Severe

bradipnea, Severe hipoxemia, Intubation and ventilation not related to anaesthesia, Oliguria non responsive to fluids or diuretic, Severe acute azotemia, Dialysis for acute renal failure, Failure to form clots, Severe acute thrombocytopenia, ive transfusion of blood or red cells, Jaundice in the presence of preeclampsia, Severe acute hyperbilirubinemia, Prolonged unconsciousness or coma, Stroke, Uncontrollable fit/status epilepticus, Global paralysis, Hysterectomy due to uterine infection or haemorrhage, Otherwise, the women has an “Infection related Maternal Death
